# Microvascular Decompression for Trigeminal Neuralgia Using Autologous Muscle Grafting: A Retrospective Analysis in a Resource-Limited Setting

**DOI:** 10.7759/cureus.81362

**Published:** 2025-03-28

**Authors:** Abdullah Alasta, Abdullah M Al_Naggar, Abdullah AL-saidy, Fuad Al Wesabi

**Affiliations:** 1 Department of Neurosurgery, Modern European Hospital, Sana'a, YEM; 2 Department of Anesthesia, Al Thawra Modern General Hospital, Sana'a, YEM

**Keywords:** autologous muscle graft, microvascular decompression, muscle graft, trigeminal neuralgia, vascular compression

## Abstract

Background

Trigeminal neuralgia (TN) is a rare and painful condition that offers various treatment options. Despite the availability of multiple modalities, their comparative efficacy is still debated due to inconsistent study outcomes. Microvascular decompression (MVD) using autologous muscle grafts has recently gained attention as a treatment option. While muscle grafts were previously employed extensively, this approach is only now being introduced in Yemen, where documented outcomes related to MVD for TN are scarce. This study aims to present the outcomes of MVD for TN utilizing autologous muscle grafts in a resource-limited environment.

Patients and methods

This study employs a retrospective cross-sectional design involving 324 patients diagnosed with TN who underwent MVD using autologous muscle grafts between April 1, 2006, and March 25, 2020. Data regarding demographic and clinical factors, outcomes, and complications were systematically collected and analyzed.

Results

The mean age of the patients was 48.8±11.6 years, with the majority being female patients (n=180, 55.6%), with a significant proportion of patients exhibiting left-sided involvement (n=204; 63%). Significant nerve compression was the primary operative finding in the study population (n=251; 77.5%). In most cases, a single vessel contributed to the compression of the nerve, primarily the superior cerebellar artery (n=303; 93.5%). No major surgical complications were reported, with temporary nasal cerebrospinal fluid (CSF) leakage (n=1; 0.3%), transient facial numbness (n=16; 4.9%) that resolved within one week to one month, hyperacusis (n=5; 1.5%), and hyperesthesia (n=3; 0.9%) being the common ones. Follow-up over an average of 52.7±8.0 months indicated that the majority of patients (n=302; 93.2%) achieved favorable outcomes, as reflected in Barrow Neurological Institute (BNI) scores I and II. The BNI pain intensity score improved from V preoperatively to I and II and was statistically significant (P=0.006). The remaining patients (n=22; 6.8%) exhibited fair to poor BNI scores (III, IV, and V). Sixteen patients obtained effective pain relief through medication, while six required radiofrequency thermocoagulation.

Conclusion

The findings suggest that MVD utilizing autologous muscle grafts may serve as an effective long-term surgical intervention for TN, even in resource-limited settings. Effective management of TN necessitates a comprehensive preoperative assessment, careful candidate selection, appropriate imaging techniques, and proficient surgical execution.

## Introduction

Trigeminal neuralgia (TN), called tic douloureux or Fothergill's disease, is a clinical syndrome characterized by episodic, unilateral, lancinating facial pain [1]. This condition is marked by discomfort in the facial region, triggered by cutaneous stimuli [2]. TN predominantly affects individuals over the age of 50, with a female-to-male prevalence ratio of 1.5:1 and an incidence rate ranging from four to 13 cases per 100,000 individuals [2,3]. The etiology of TN is primarily caused by vascular compression. Other potential causes include multiple sclerosis, tumor-induced nerve compression, arteriovenous malformations, and trauma to the trigeminal nerve resulting from procedures such as sinus surgery, oral surgery, strokes, or facial injuries [4].

TN remains one of the few neuropathic pain syndromes that can be managed through conservative, pharmacological, and surgical interventions [4-6]. However, the optimal therapeutic approach has yet to be definitively established [6]. The complexities involved in identifying the most effective treatment for TN arise from the necessity for clearly defined outcome measures. Consequently, comparative assessments of the therapeutic modalities pose significant challenges [3,6]. Among the available surgical interventions, percutaneous techniques, gamma knife surgery (GKS), and microvascular decompression (MVD) are the primary strategies currently employed. MVD has gained popularity due to its efficacy, relative safety profile, and minimal, yet manageable, neurological sequelae [7,8]. Although MVD neurosurgery has proven effective in alleviating TN by resolving the conflict between the trigeminal nerve and the vertebrobasilar artery (VBA), the long-term outcomes regarding pain relief and procedural risks remain insufficiently clarified. 

The incidence and severity of complications associated with MVD are primarily influenced by the surgeon's skill and familiarity with the local anatomy, as well as the dimensions and configuration of the implants used [9,10]. Various synthetic materials, including Teflon, Ivalon, Gelfoam, and Gore-Tex, have been utilized during MVD procedures [10,11]. Despite their widespread application, existing literature has reported complications such as granuloma formation and chemical meningitis [12,13]. In contrast, some surgeons have noted comparable outcomes when using autologous muscle grafts (AMG) and the potential for reduced complication rates [7,14]. However, there is a lack of empirical evidence regarding the use of AMG in MVD for TN. This is specifically regarding their efficacy and complication rates compared to synthetic materials. This knowledge gap hinders a comprehensive understanding of the viability and benefits of AMG in this patient population [15]. 

The implementation of MVD for TN is still in its early stages in low-income countries, primarily due to insufficient healthcare funding, a shortage of skilled practitioners, and the absence of an academic framework dedicated to this complex surgical technique [15,16]. The objective of the present study is to document the therapeutic outcomes of MVD for TN utilizing AMG within a resource-constrained environment. To our knowledge, this investigation represents the first study of its kind in Yemen.

## Materials and methods

Study design

This retrospective cohort study included patients diagnosed with classical TN according to the International Classification of Headache Disorders, 3rd edition beta (ICDH-3 beta) [17], who underwent MVD using AMG at the Neurosurgery Department of Modern European Hospital in Sana'a, Yemen, between April 1, 2006, and March 25, 2020. The MVD procedures were conducted by a single experienced neurosurgeon, Dr. Alasta. A total of 324 operations were performed during this period. The study received ethical approval from the ethics committee of Modern European Hospital and was conducted in accordance with the Helsinki Declaration. Informed consent was obtained for data collection and the use of photographs from all the eligible participants.

Inclusion and exclusion criteria

The inclusion criteria for this study were adult patients diagnosed with TN based on the ICHD. Eligible patients exhibited symptoms refractory to medical treatment, minimal pain relief from previous interventions, or adverse effects that precluded medication tolerance. The diagnosis was confirmed through a thorough clinical evaluation by a neurologist, who utilized patient history and clinical examination to exclude other causes of facial pain. Preoperative assessment included high-resolution brain magnetic resonance imaging (MRI) to evaluate vascular compression. Patients who had previously failed other treatment modalities, such as radiofrequency lesioning and alcohol rhizotomy, were also included.

Patients were excluded from the study if they presented with atypical facial pain, had other TN-related pathologies, or were treated with materials other than AMG during MVD.

Preoperative assessment and surgical procedure

**Figure 1:**
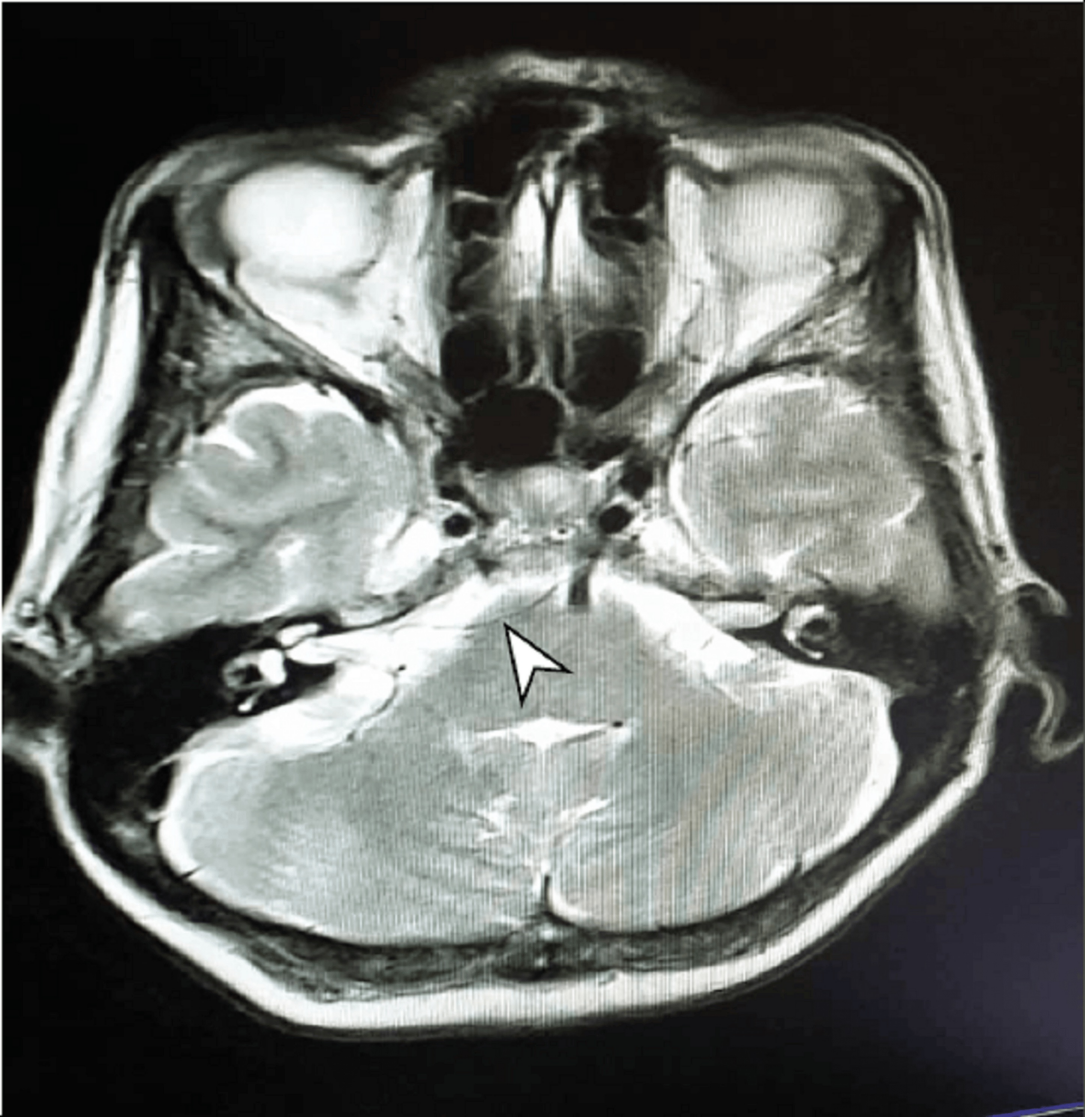
The vascular loop is observed on the T2 axial view magnetic resonance image (indicated by the arrowhead)

All patients underwent a comprehensive clinical assessment and brain MRI utilizing T2 constructive interference in steady state (CISS) sequences before the surgery (Figure 1). In certain cases, a Fast Imaging Employing Steady-state Acquisition (FIESTA) sequence was utilized. This imaging procedure aimed to identify the compressing vascular loop while ruling out the presence of brain neoplasms, vascular anomalies, and multiple sclerosis.

**Figure 2:**
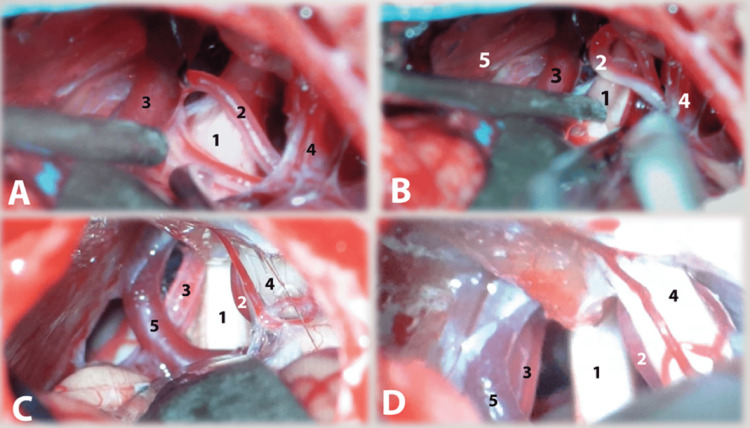
Microscopic visualization of the vascular loop compression implicated in cases of trigeminal neuralgia (A) The AICA is connected to the fifth cranial nerve. (B) The effects of compression are evident on the surface of AICA after release from the Vth nerve. (C) The SCA loop is visible on the opposite side of the Vth nerve, compressing the Vth nerve at its root entry zone. The AICA is located beneath the seventh and eighth cranial nerve complex. (D) The site is prepared for the autologous muscle graft on both sides of the trigeminal nerve. 1. Trigeminal nerve (Vth cranial nerve); 2. Anterior inferior cerebellar arteries (AICA); 3. Superior cerebellar arteries (SCA); 4. Seventh and eighth cranial nerve complex; 5. Petrosal veins.

A skilled neurosurgeon established the diagnosis of TN through a combination of clinical evaluation, laboratory analyses, and radiological imaging. All the preoperative assessments and surgical procedures were conducted by Dr. Alasta, a neurosurgeon with 15 years of experience. After the induction of general anesthesia and positioning of the patient in the supine position, a conventional retromastoid suboccipital craniectomy incision was initiated, and muscle tissue was meticulously separated from the suboccipital region. Subsequently, a 1.5 cm craniectomy was performed. The dura mater was incised and the cerebrospinal fluid (CSF) was gradually released. The trigeminal cistern was accessed through a gentle retraction of the cerebellum. The arachnoidal openings were sufficiently large to permit the exposure of the trigeminal nerve. The nerve was thoroughly examined in its entirety, extending from the root entry zone to the cisternal segment and into the parapetrous area (Figures 2A-2D).

**Figure 3:**
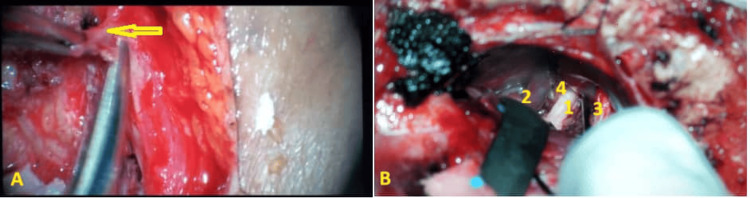
Microscopic visualization of vascular loop compression implicated in cases of trigeminal neuralgia (A) A muscle graft is interposed between the loop of the superior cerebellar artery (SCA) and the trigeminal nerve, as well as between the loop of the anterior inferior cerebellar artery (AICA) and the trigeminal nerve on the contralateral side (yellow arrow). (B) Autologous muscle graft effectively spans the entire length of the trigeminal nerve, from its emergence from the brainstem to its root entry zone. 1. Trigeminal nerve (Vth cranial nerve); 2. Petrosal veins; 3. Interposition of a muscle graft between the anterior inferior cerebellar artery (AICA) and the trigeminal nerve; 4. Interposition of muscle grafts between the spinal accessory nerve and the trigeminal nerve

Delicate microscopic dissection techniques were employed to delineate and isolate the associated arteries and/or veins. Muscle tissue served as a protective barrier between the nerve and the vascular structures. A segment of muscle was interposed between the offending blood vessel and the root entry zone. No fibrin glue or sutures were used to immobilize the muscle (Figures 3A-3B).

A watertight closure of the dura was achieved, with or without the application of autologous fascia, utilizing continuous suturing techniques with 4-0 Vicryl round-bodied sutures (Ethicon, Johnson & Johnson, Somerville, NJ, USA). The closure of the wound was performed in anatomical layers, specifically involving continuous suturing of the muscle layer, fascial suturing where preserved, interrupted suturing of the subcutaneous layer, and final suturing of the skin.

Postoperative care and follow-up outcomes

The same surgeon conducted the early postoperative examination and assessed the postoperative state of the cranial nerve function. Telephonic interviews were utilized for additional follow-up regarding the patient's condition. The Barrow Neurological Institute's (BNI) pain intensity score was employed to quantify pain severity [18]. A BNI score of 0 indicates that no medication was required at the time of testing, while a score of I signifies an excellent effect: no pain. A BNI score of II indicates a good effect: occasional pain that does not, or only occasionally, diminishes quality of life. A BNI score of III reflects a limited effect: daily pain with a moderate reduction in quality of life. Finally, a BNI score of IV denotes an insufficient effect: daily episodes of severe pain that significantly impair quality of life (Table 1).

**Table 1:** Barrow Neurological Institute pain intensity score

Score	Description of pain relief
I	No pain; no medication required
II	Occasional pain; does not necessitate medication
III	Some pain; effectively managed with medication
IV	Some pain; not sufficiently managed by medication
V	Severe pain; no relief provided by medication

Data collection

Demographic factors, including age, gender, duration and location of pain, and comorbidities such as diabetes mellitus and hypertension, were recorded. Additional information gathered included previous medical treatments and their durations, prior surgical interventions, MRI findings, surgical details such as the number of involved arteries and their duration, surgical outcomes, postoperative complications, follow-up data, treatment failures, and time to recurrence. The collected data underwent a thorough review for accuracy, completeness, and consistency. In cases where contradictory or missing information was identified, medical charts were reexamined and reevaluated to ensure data integrity.

Main outcome

The primary objective of this study was to evaluate the outcomes of MDV using AMG, as measured by the BNI score, along with the assessment of postoperative complications and recurrence rates.

Statistical analysis

We utilized mean values and standard deviations (SD) to present numerical data, while frequencies and percentages were employed to represent qualitative data. A chi-square test was conducted to compare categorical data, and the Student's t-test was performed to assess differences between two independent groups. A P-value of less than 0.05 was deemed statistically significant. All statistical analyses were carried out using IBM SPSS Statistics for Windows, Version 22 (Released 2013; IBM Corp., Armonk, New York, United States). 

## Results

The mean age of the patients was 48.8±11.6 years, ranging from 29 to 69 years. The cohort comprised more female patients (n=180; 55.6%) than male ones (n=144; 44.4%). More than half of the TN cases (n=204, 63.0%) involved the left side. The majority of patients reported pain in both the maxillary and mandibular branches of the trigeminal nerve (n=160, 49.4%), followed by pain exclusively in the maxillary branch (n=127, 39.2%), while some experienced pain in both the ophthalmic and maxillary branches (n=37, 11.4%). Most patients predominantly described the pain as a sensation of the skin being injected with a red-hot needle (n=199, 61.4%). Symptoms lasted an average of 25.6±8.5 months, with a range of 12.0 to 45.0 months. Some patients reported a family history of neurological disease (n=21; 6.5%). Hypertension (n=120; 37%) and prior neurodestructive surgical procedures (n=9; 2.8%) were also noted. Before undergoing MVD, all the patients were receiving medications specifically for TN, with a majority of them on carbamazepine (n=160; 49.4%) and some on other medications (n=127 patients; 39.2%) to manage their symptoms. Table 2 presents the characteristics of patients with TN who underwent MVD.

**Table 2:** Characteristics of the study population

Variable	Overall (n=324)
Age (years), Mean±SD	48.8 ± 11.6 (Range: 29-69)
Gender	
Male	144 (44.4%)
Female	180 (55.6%)
Location of the pain	
Both maxillary and mandibular trigeminal nerve	160 (49.4%)
Maxillary trigeminal nerve alone	127 (39.2%)
Both ophthalmic and maxillary trigeminal nerve	37 (11.4%)
Affected side	
Left	204 (63.0%)
Right	120 (37.0%)
Previous medical treatment	
Oxcarbazepine	37 (11.4%)
Carbamazepine	160 (49.4%)
More than one drug	127 (39.2%)
Duration of symptoms (months), Mean±SD	25.6±8.5 (Range: 12-45)
Comorbidities	
Family history of neurologic disorders	21 (6.5%)
Hypertension	120 (37.0%)
Previous neurodestructive surgery	9 (2.8%)
Pain sensation	
Injection-like pain (e.g., feeling of being injected with a red-hot needle)	199 (61.4%)
Burning	120 (37.0%)
Sharp and shooting	115 (35.5%)
Electric shock-like	87 (26.9%)
Mixed quality	12 (3.7%)

Radiologic and operative characteristics

The MRIs revealed ipsilateral nerve compression in most patients (n=303; 93.5%). Other findings included bilateral nerve compression (n=9; 2.8%), no or doubtful compression (n=9; 2.8%), and contralateral nerve compression (n=3; 0.9%). The primary operative finding was significant nerve compression (n=251; 77.5%). In most instances, a single vessel was compressed (n=302; 93.2%), with the superior cerebellar artery identified as the main compressing vessel (n=303; 93.5%). Multiple nerve branches were involved in most cases (n=206; 63.6%). All patients exhibited neurovascular conflict and subsequently underwent MVD using AMG (Table 3).

**Table 3:** Radiologic and operative characteristics of the study population

Variable	Overall (n=324)
Magnetic resonance imaging	
Ipsilateral nerve compression	303 (93.5%)
Contralateral nerve compression	3 (0.9%)
Bilateral nerve compression	9 (2.8%)
No/doubtful compression	9 (2.8%)
Operative findings	
Significant nerve compression	251 (77.5%)
Thick arachnoid membrane	52 (16.0%)
Narrow cisterna	2 (0.6%)
Small posterior fossa	9 (2.8%)
Mild compression	9 (2.8%)
No contact	1 (0.3%)
Compressing vessel	
Superior cerebellar artery	303 (93.5%)
Inferior	3 (0.9%)
Basilar	9 (2.8%)
Other vessels	9 (2.8%)
Number of compressed vessels	
One	302 (93.2%)
More than one vessel	22 (6.8%)
Number of nerve branches	
Single	118 (36.4%)
Multiple	206 (63.6%)

Postoperative complications and follow-up outcomes

No significant complications were identified during the surgical intervention, with only one patient (0.3%) experiencing a transient cerebrospinal fluid (CSF) leak through the nasal cavity. The condition was managed conservatively, with the patient placed on bed rest and observed for one day, during which the symptoms improved. Some patients (n=3; 0.9%) presented with pre-existing facial paresthesia, which showed no significant change following the surgical procedure; therefore, these cases were not classified as operative complications. Transient facial paresthesia was observed in some patients (n=16; 4.9%), but it resolved within one week to one month. Instances of hyperacusis were reported by a few patients (n=5; 1.5%), while an additional number of patients (n=3; 0.9%) experienced hyperesthesia. A follow-up assessment, with a mean duration of 52.7±8.0 months (ranging from 33.0 to 65.0 months), revealed that most patients (n=302; 93.2%) achieved favorable outcomes, as indicated by the BNI scores of I and II. The BNI pain intensity score improved from V preoperatively to I and II, demonstrating statistical significance (P=0.006). The remaining patients (n=22; 6.8%) exhibited fair to poor BNI scores of III, IV, and V. In this subset, the majority of patients (n=16) reported satisfactory pain relief through pharmacological means, while the rest (n=6) required radiofrequency thermocoagulation to manage their symptoms (Table 4).

**Table 4:** Postoperative complications and follow-up outcomes in the study population CSF: Cerebrospinal fluid; BMI: Barrow Neurological Institute

Variable	N (%)
Postoperative complications	
Temporary leakage of CSF through the nasal passage	1 (0.3%)
Facial numbness	3 (0.9%)
Transient facial numbness	16 (4.9%)
Hyperacusis	5 (1.5%)
Hyperesthesia	3 (0.9%)
Follow-up time (months), Mean±SD	52.7±8.0 (Range: 33.0-65.0)
Outcome	
Positive outcomes (BNI scores I and II)	302 (93.2%)
Negative outcomes (BNI III, IV, and V)	22 (6.8%)

## Discussion

TN is a chronic pain disorder affecting the trigeminal nerve, which transmits sensations from the face to the brain. The condition is often treated with medications; however, these may not provide satisfactory relief for all patients. MVD is a surgical procedure that has become a viable alternative for the treatment of TN [15]. In this study, we investigated the therapeutic outcomes and postoperative complications in patients with TN treated using MVD with AMG. Our study revealed that this is a reliable procedure for alleviating TN symptoms, yielding positive functional results even in resource-limited settings. Notably, most participants (n=302; 93.2%) achieved BNI scores classified as I and II post-MVD. Furthermore, the overall complication rate remained low and was comparable to the figures reported in previous literature [15,19,20].

The average duration of TN symptoms in our study was 25.6 months (range: 12-45 months), aligning with existing studies that report a mean duration of 24.7 months before MVD [15,19,21]. Conversely, Louges et al. documented an average interval of seven years between the initial administration of carbamazepine and subsequent MVD [2]. This extended delay results in significant discomfort for patients experiencing prolonged pain and may stem from inadequate awareness among neurologists regarding the availability, effectiveness, and appropriate indications for MVD. Consistent with prior research, we found that the superior cerebellar artery was the predominant vascular structure involved in neurovascular compression [2,21].

The success of postoperative outcomes can be attributed to various factors, including optimal patient positioning [22]. In this study, we maintained a supine-oblique position for the patients with sufficient head and neck support, which is optimal for surgeries on the posterior fossa lesions. This position enhances stability and surgical accessibility while allowing for close patient monitoring, consistent with established physiological norms [23]. Additionally, meticulous intraoperative management strategies, such as limited skin incisions and careful dural closure, significantly influence the outcomes of microvascular decompression and recovery. Walter Dandy's U-shaped incision technique for craniotomy exposure is noteworthy [24], as it minimizes the risk of injury to the occipital artery and nerve while also facilitating reduced muscle dissection. Our vertical linear retrosigmoid incision improves surgical exposure and decreases craniectomy size, resulting in aesthetically favorable outcomes, as reported by Nurimanov et al. [23].

In our study, craniectomies were performed with an average surface area of 4 cm² (20 mm x 20 mm) using bone rongeurs. This method is posited to reduce the incidence of CSF leakage by lowering the likelihood of mastoid cell exposure [23,25]. Remarkably, only one patient in our cohort experienced CSF leakage, leading to an incidence rate of 0.3%, considerably lower than rates documented in other studies, ranging from 2.3% to 2.8% [20,26]. CSF leakage is concerning due to its association with prolonged hospitalization, increased infection risk, and elevated treatment costs [23,27]. Furthermore, we typically do not utilize lumbar catheters or Jannetta's retractors for CSF drainage. Instead, we employ microsurgical approaches to access the basolateral cisterns, facilitating natural CSF drainage while minimizing complications and ensuring excellent visualization of neurovascular structures, as noted by Nurimanov et al. [23].

Preserving the superior petrosal vein (SPV) is critical when approaching the trigeminal nerve. While some practitioners recommend dissection and coagulation of the SPV, others advocate for its preservation to maintain normal anatomical configurations [28-30]. Our primary objective has been to uphold the integrity of these vital structures, ensuring the preservation of the SPV complex in all the cases.

A pivotal aspect of MVD involves identifying and mobilizing neurovascular conflicts between vascular structures and neural pathways [23]. Venous compression affecting the trigeminal nerve is associated with a higher likelihood of recurrence compared to cases of arterial compression. Research conducted by Nair et al. indicates that patients with TN resulting from venous compression generally experience more pronounced pain outcomes post-MVD compared to those affected solely by arterial compression [30]. In our demographic analysis, cases characterized by suboptimal and fair outcomes may correlate with venous compression. In such instances, gamma-knife radiosurgery is suggested as a therapeutic alternative. It is crucial to identify additional indicators of compression, such as nerve atrophy, arterial impressions, grooving, and deviations in the nerve's trajectory, as some neurovascular conflicts may not present as TN [31].

MVD employs various materials, each with distinct outcomes and associated complications. Teflon is a commonly-used synthetic option due to its inertness. However, it has been linked to recurrence rates of 12% (0-30%) and complications 12% (0-30%) such as granuloma formation and chemical meningitis [12,13,32]. In contrast, AMG demonstrate a superior success rate of 93%, with lower recurrence rates and complications. Our findings indicate that patients who received AMG had fewer serious adverse events compared to those treated with synthetic materials in other reports. However, there are still concerns about its association with a higher long-term recurrence rate. Jagannath et al. reported that the autologous muscle provided complete pain relief in 78% of patients and significant relief in 21.2%, although their selection criteria were not detailed [7]. Additionally, Ashraf et al. highlighted that the use of autologous muscle yielded long-term relief for TN with vascular compression and minimal morbidity, establishing it as a preferable alternative to synthetic materials [15]. While concerns regarding muscle resorption persist, utilizing larger muscle segments can help mitigate these issues during surgery. Moreover, the design of expanded polytetrafluoroethylene (ePTFE) sleeves offers advantages, such as improved nerve isolation from blood vessels, potentially enhancing clinical outcomes. This is based on previous research on hemifacial spasms [33]. A summary of the materials used in MVD and their associated outcomes is provided in Table 5.

**Table 5:** Summary of the materials used in microvascular decompression (MVD) and their associated outcomes in previously published reports MVD: Microvascular Decompression; TN: Trigeminal Neuralgia; N/A: Not Applicable; CSF: Cerebrospinal Fluid; AMG: Autologous Muscle Grafts. ^*^Indicates the percentage of patients requiring additional medication for pain control; ^†^Denotes the occurrence of specific complications requiring treatment; ^‡^Reflects the results of the recent study's recurrence rates; ^§^Indicates variability in recurrent trends based on treatment methods; ^||^Highlights the absence of recorded complications in the specified study; ^¶^There were no recorded complications one year after the surgery; ^††^Indicates variability in outcomes and complications among grafts; ^‡‡^Reflects a significant percentage of complications related to the surgical procedure; ^§§^Highlights notable findings regarding long-term effectiveness.

Study (Year)	Material used	Recurrence rate	Time of recurrence	Complication rate	Comments
Paolini et al. (2025) [1]	AMG	3.5%^*^	6 months	5.3% needed medication^†^	AMG graft provided immediate pain relief with low recurrence.
Peng et al. (2024) [34]	Teflon	11.3%^‡^	12 months	N/A	Developed a nomogram for predicting recurrence based on various clinical features.
Ashraf et al. (2024) [15]	AMG	8-12%^§^	12-18 months	12.10%	Graft viability impacted outcomes; some complications were noted.
Amagasaki et al. (2024) [5]	Teflon	27.80%	12 months	No complications	1-2 years
Liu et al. (2021)^||^ [35]	Teflon (Traditional vs. MVD plus)	3.4% vs. 0.55%^¶^	6 months in MVD and 12 months in MVD plus	N/A	MVD plus showed improved outcomes due to supplementary neurolysis.
Zheng et al. (2019) [36]	Teflon	9% at one year	12 months	N/A	Factors influencing recurrence identified.
Jagannath et al. (2018) [7]	AMG	13.9%^††^	6-24 months	16.1%^‡‡^	Minimal complications reported with risks of scarring.
Chen et al. (2000) [13]	Teflon	11.2%^§§^	3 years	5.6% teflon granuloma	Explored long-term outcomes over three years.

Upon nerve decompression, we implemented the 'MVD plus' technique, incorporating intraoperative neurolysis. Findings from Liu et al. suggest that the combination of adequate MVD with nerve-sparing techniques yields superior cure rates and reduced recurrence compared to MVD performed in isolation [35]. We hypothesize that the employment of precise methodologies - including miniature skin incisions, limited craniotomies, the MVD plus technique, and AMG - not only minimizes the risk of CSF leakage but also enhances postoperative aesthetic outcomes and achieves commendable BNI scores, despite the lack of overt neurovascular conflicts.

A previous case series revealed that 80.8% of 26 patients with mixed TN experienced significant pain relief with AMG [37]. Additionally, another study documented that 91.8% of participants achieved partial or complete relief, with 86.3% attaining complete pain relief, which mirrors our cohort’s finding of 93.2% experiencing similar relief [38]. The current study's postoperative complication rate was notably low, aligning with existing research [2,3]. Thus, we reported no cases of mortality, peripheral facial palsy, hypoesthesia, or permanent cochleovestibular damage. The short length of hospital stay solidifies the view of microsurgical vascular decompression as a safe treatment option across all ages when performed by adept professionals. In our cohort, 22 patients (6.8%) had fair to poor BNI scores (III, IV, and V), with 16 of them obtaining satisfactory pain relief through medication and six requiring radiofrequency thermocoagulation. Similarly, Louges et al. indicated a 10.3% overall failure rate and a 12.6% recurrence rate, including two patients who underwent successful revision [2]. Failures in this series were infrequent and primarily occurred within two years, with increased recurrence risk linked to factors such as non-use of endoscopy, the origins of venous compression, polytetrafluoroethylene application, and previous treatment history [2].

Study limitations

This study has several significant limitations. Notably, it relies on secondary data, which may vary in quality due to inconsistencies in documentation. The retrospective, single-center design introduces potential biases, including selection, information, and measurement biases, as outcomes were assessed by the surgeon who performed the procedures. Furthermore, the study did not address how factors such as location, lifestyle, education level, depression, anxiety, or other comorbid conditions may affect postoperative pain improvement. The follow-up period was relatively short, potentially leading to a higher recurrence rate, and comparisons with other materials were not made, limiting conclusions on the efficacy of AMG. Additionally, the lack of a comparative statistical analysis of predictors for recurrence or treatment failure weakens the findings. The follow-up methodology, primarily based on telephonic interviews, may introduce recall bias, particularly for subjective outcomes like pain, and some patients may have been unreachable, limiting generalizability. Despite these limitations, the study benefits from a large sample size. However, the absence of a control group restricts conclusions regarding treatment efficacy. Future research should involve multi-center designs, larger sample sizes, and more robust follow-up methodologies, including in-person assessments and standardized questionnaires, to improve the reliability and applicability of these findings in the management of TN.

## Conclusions

Our data suggest that MVD using AMG can be an effective long-term surgical intervention for TN, even in resource-limited settings. The management of TN can be successfully achieved through careful preoperative evaluation, candidate selection, appropriate imaging techniques, and skilled surgical intervention. Moreover, factors such as patient positioning and intraoperative techniques such as minor skin incisions, limited craniotomy, precise dural closure, and intraoperative neurolysis may play a significant role in achieving favorable clinical outcomes and satisfactory postoperative aesthetics. While our findings indicate a favorable outcome rate (BNI scores I and II), based on the collected data, further research is warranted to compare the efficacy of AMG with alternative materials and to assess their long-term effectiveness.
